# Supplementary material for: Urinary Titin N-Fragment Evaluation in a Randomized Controlled Trial of Beta-Hydroxy-Beta-Methylbutyrate for Acute Mild Trauma in Older Adults
Source: Nutrients. 2021 Mar 10;13(3):899. doi: 10.3390/nu13030899 (PMC8001392; doi:10.3390/nu13030899)
Supplement: Supplementary file 1 [file nutrients-13-00899-s001.pdf]

Supplementary Materials

Urinary titin N-fragment evaluation in a randomized controlled trial of beta-hydroxy-beta-methylbutyrate for acute mild trauma in older adults

Table S1. Comparison between groups among patients with follow-up evaluation during hospitalization

	HMB	GFO	p
**n**	20	15	NA
**Age, years**	84.2 (5.0)	86.5 (7.3)	0.271
**Male , n(%)**	9 (45.0)	6 (40.0)	1
**SOFA maximum**	3.0 [2.0 to 4.2]	4.0 [3.0 to 5.0]	0.114
**APACHE II**	10.0 [8.0 to 12.5]	12.0 [9.5 to 18.0]	0.248
**CCI**	2.0 [1.0 to 4.0]	1.0 [1.0 to 2.0]	0.221
**ISS**	4.0 [4.0 to 9.0]	9.0 [7.5 to 9.0]	0.156
**Head and neck**	4 (20.0)	4 (26.7)	0.954
**Face**	0 (0.0)	1 (6.7)	0.884
**Chest**	6 (30.0)	4 (26.7)	1
**Abdomen**	5 (25.0)	3 (20.0)	1
**Extremities and pelvis**	7 (35.0)	6 (40.0)	1
**Surface**	20 (100.0)	15 (100.0)	NA
**Length of hospitalization, day**	25.0 [16.8 to 43.0]	23.0 [15.5 to 29.0]	0.433
**ICU admission, n(%)**	3 (15.0)	0 (0.0)	0.338
**Death, n(%)**	1 (5.0)	1 (6.7)	1
**Follow up evaluation day**	13.8 (2.1)	13.6 (2.3)	0.735
**RFCSA**			
**pre, mm2**	2.4 (0.8)	2.2 (0.6)	0.402
**post, mm2**	2.3 (0.8)	2.3 (0.8)	0.94
**change, mm2**	-0.0 [-0.6 to 0.4]	0.2 [-0.1 to 0.4]	0.342
**Grip strength**			

**pre, kg**	10.9 (7.1)	8.4 (6.7)	0.286
**post, kg**	11.0 (7.9)	10.2 (7.1)	0.764
**change, kg**	0.0 [-1.3 to 2.7]	0.0 [-0.6 to 2.2]	0.726
**Barthel Index**			
**pre**	100.0 [90.0 to 100.0]	100.0 [90.0 to 100.0]	0.651
**post**	15.0 [8.8 to 56.2]	40.0 [12.5 to 57.5]	0.639
**change**	-62.5 [-85.0 to -28.8]	-50.0 [-75.0 to -32.5]	0.688
**N-titin/Cre**			
**day1, pmol/mgCre**	22.3 [15.6 to 26.7]	23.1 [16.4 to 39.4]	0.536
**day3, pmol/mgCre**	26.6 [18.9 to 40.3]	20.2 [14.1 to 28.9]	0.423
**change, pmol/mgCre**	5.9 [0.9 to 21.0]	-0.4 [-5.7 to 13.1]	0.161

Data were shown as means ± SD or medians [IQR]. HMB = beta-hydroxy-beta-methylbutyrate; RFSCA = cross-sectional area of the rectus femoris, N-titin/Cre = spot urine titin N-fragment divided by spot urine creatinine multiplied by 10.

Table S2. Comparison between groups of patients with outpatient follow-up evaluation

	HMB	GFO	p
**n**	4	10	NA
**Age, years**	81.5 (6.0)	82.4 (5.8)	0.8
**Male , n(%)**	3 (75.0)	4 (40.0)	0.554
**SOFA maximum**	4.0 [1.5 to 6.2]	2.5 [1.0 to 4.0]	0.515
**APACHE II**	10.0 [8.8 to 11.0]	8.5 [8.0 to 10.8]	0.565
**CCI**	0.5 [0.0 to 1.2]	1.0 [1.0 to 2.8]	0.272
**ISS**	2.5 [1.0 to 5.2]	8.5 [4.0 to 9.0]	0.216
**Head and neck**	3 (75.0)	5 (50.0)	0.798
**Face**	0 (0.0)	0 (0.0)	NA
**Chest**	0 (0.0)	3 (30.0)	0.607
**Abdomen**	0 (0.0)	0 (0.0)	NA
**Extremities and pelvis**	1 (25.0)	2 (20.0)	1

**Surface**	0 (0.0)	0 (0.0)	NA
**Length of hospitalization, day**	6.0 [4.5 to 7.0]	4.0 [3.2 to 5.8]	0.565
**ICU admission, n(%)**	4 (100.0)	10 (100.0)	NA
**Death, n(%)**	4 (100.0)	10 (100.0)	NA
**Follow up evaluation day**	18.8 (4.6)	17.4 (2.5)	0.479
**RFCSA**			
**pre, mm2**	2.9 (0.7)	2.4 (0.6)	0.246
**post, mm2**	3.2 (1.3)	2.7 (1.5)	0.587
**change, mm2**	0.3 [-0.5 to 1.1]	0.4 [-0.1 to 0.9]	1
**Grip strength**			
**pre, kg**	20.1 (1.4)	13.4 (6.7)	0.075
**post, kg**	24.7 (4.9)	17.4 (9.9)	0.196
**change, kg**	3.4 [0.9 to 7.1]	2.5 [0.5 to 7.7]	0.777
**Barthel Index**			
**pre**	100.0 [100.0 to 100.0]	100.0 [100.0 to 100.0]	0.353
**post**	100.0 [100.0 to 100.0]	95.0 [60.0 to 100.0]	0.06
**change**	0.0 [0.0 to 0.0]	-5.0 [-10.0 to 0.0]	0.06
**N-titin/Cre**			
**day1, pmol/mgCre**	27.5 [18.2 to 37.6]	20.6 [11.7 to 26.8]	0.355
**day3, pmol/mgCre**	29.8 [25.2 to 34.4]	13.0 [8.7 to 17.7]	0.079
**change, pmol/mgCre**	-1.1 [-8.7 to 5.2]	1.0 [-3.0 to 2.6]	0.782

Data were shown as means ± SD or medians [IQR]. HMB = beta-hydroxy-beta-methylbutyrate; RFSCA = cross-sectional area of the rectus femoris, N-titin/Cre = spot urine titin N-fragment divided by spot urine creatinine multiplied by 10.

Figure S1 Correlation of urinary creatinine with N-titin/Cre and muscle injury endpoints

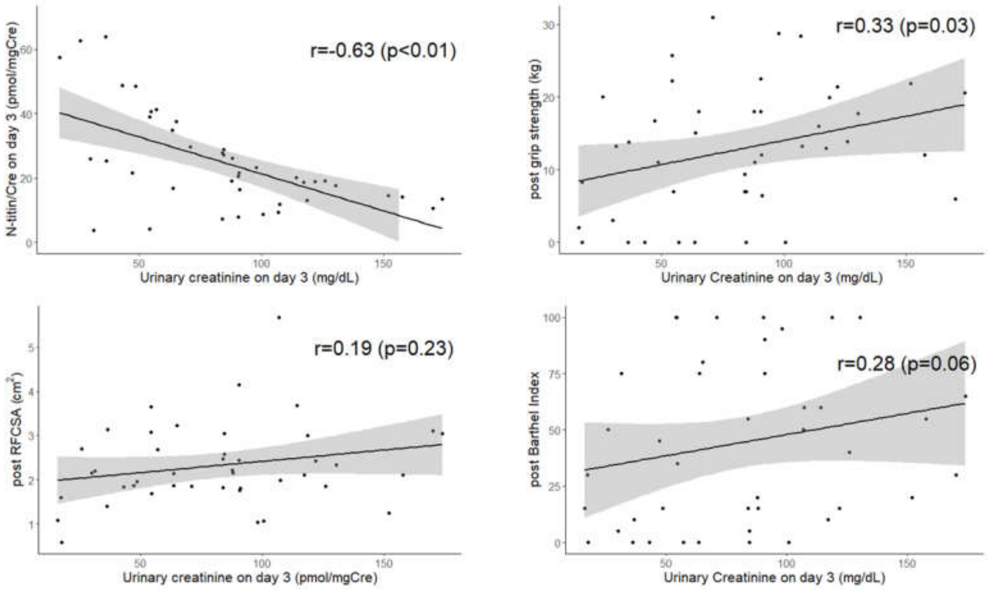

In scatter plots, the regression line is shown as a solid line, with 95% confidence intervals as shaded areas. Comparison of N-titin/Cre and urinary creatinine on day 3 showed an inverse correlation ($r=-0.63$, $p<0.01$), but the correlation between urinary creatinine and the muscle injury endpoints was inferior to that between N-titin/Cre and the muscle injury endpoints.

RFCSA = cross-sectional area of the rectus femoris; N-titin/Cre = spot urine titin N-fragment divided by spot urine creatinine multiplied by 10.
